# Childhood trauma fatality and resource allocation in injury control programs in a developing country

**DOI:** 10.1186/1471-2458-6-117

**Published:** 2006-05-02

**Authors:** Bahman S Roudsari, Mazyar Shadman, Mohammad Ghodsi

**Affiliations:** 1 Harborview Injury Prevention and Research Center and Department of Epidemiology, University of Washington, Seattle, WA, USA; 2 Sina Trauma and Surgery Research Center, Tehran University of Medical Sciences, Tehran, Iran

## Abstract

**Background:**

Only a few studies have addressed the trimodal distribution of childhood trauma fatalities in lesser developed countries. We conducted this study to evaluate pre-hospital, Emergency Department (ED) and in-hospital distribution of childhood injury-related death for each mechanism of injury in Tehran, Iran. This information will be used for the efficient allocation of the limited injury control resources in the city.

**Methods:**

We used Tehran's Legal Medicine Organization (LMO) database. This is the largest and the most complete database that receives information about trauma fatalities from more than 100 small and large hospitals in Tehran. We reviewed all the medical records and legal documents of the deceased registered in LMO from September 1999 to September 2000. Demographic and injury related characteristics of the children 15 years old or younger were extracted from the records.

**Results:**

Ten percent of the 4,233 trauma deaths registered in LMO occurred among children 15 years old or younger. Motor vehicle crashes (MVCs) (50%), burns (18%), falls (6%) and poisonings (6%) were the most common mechanisms of unintentional fatal injuries. Prehospital, emergency department and hospital deaths comprised 42%, 20% and 37% of the trauma fatalities, respectively. While, more than 80% of fatal injuries due to poisoning and drowning occurred in prehospital setting, 92% of burn-related fatalities happened after hospital admission.

**Conclusion:**

Injury prevention is the single most important solution for controlling trauma fatalities due to poisoning and drowning. Improvements in the quality of care in hospitals and intensive care units might substantially alleviate the magnitude of the problem due to burns. Improvements in prehospital and ED care might significantly decrease MVC and falls-related fatalities.

## Background

Traditionally infectious diseases have been the leading cause of death especially among children [1]. However with the efforts of local health departments and international organizations such as World Health Organization, infectious diseases have been substantially controlled and injuries have become the number one cause of death in children and young adults in many high income countries [2]. Similar transition has been started in many lesser-developed countries [2]. It is predicted that by 2020 trauma will be the first or second leading cause of death for both developed and developing nations [3]. The high proportion of children and young adults and the substantial socio-economic consequences of childhood injuries in lesser developed countries [4] require prudent attention to the issue of injury control.

**Figure 1:**
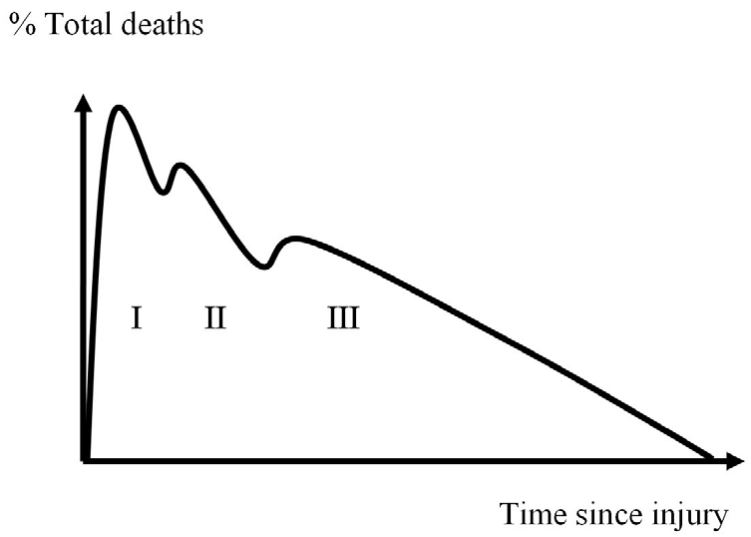
Trimodal distribution of fatal injuries.

Figure 1 shows that trauma fatality follows a trimodal distribution [5,6]. In Phase I, death occurs immediately or quickly as a result of overwhelming injury [5]. Due to the severity of injury and short time interval between injury and death, patients often do not have the opportunity for benefiting from medical interventions. Therefore, improvements in quality of care does not significantly influence patients' outcome [5,6]. Phase II includes fatalities that occur within several hours of the event [5]. These injuries are often less severe and leave more time for prehospital and hospital care providers to intervene and improve patients' outcome. Under these circumstances, improvements in the quality of prehospital, and ED care can alleviate the magnitude of trauma fatality [5,6]. Deaths in Phase III, occur days or weeks after injury. These fatalities often occur in a hospital. Therefore patients' outcome is highly correlated with the quality of care that they receive in hospitals or Intensive Care Units (ICUs).

Identification of the relative importance of each phase of trauma fatality for any particular mechanism of injury has a substantial value for injury control programs. Based on this information, public health advocates and policy makers can allocate the limited injury control resources among prevention activities and prehospital and hospital care improvement programs more efficiently.

Currently very limited injury prevention activities have targeted children in Tehran. As the first step in controlling childhood injuries in our community, we evaluated the demographic and injury related characteristics of different mechanism of injury among hospitalized children [7]. However significant proportion of fatal injuries in developing countries occur in prehospital settings [1] which are not captured in hospital databases. We conducted this study in order to identify the trimodal distribution of trauma fatality for each mechanism of injury. We are hopeful that the results of this study help us prioritize our future injury control programs.

## Methods

### Settings

Tehran is a city with more than 12 million populations. There are more than 100 small and large hospitals that provide different levels of care to trauma victims. Currently there is no established trauma system in the city and no hospital has been designated as a Level I trauma center. However, a few university hospitals play the role of the tertiary medical care facilities.

Prehospital trauma care is provided by Tehran's emergency medical service (EMS) system [8]. Approximately 15% of the trauma patients are transported to a medical care facility by an EMS ambulance [7,9]. The city has been divided into 22 regions and each region has an ambulance station. In the absence of a designated trauma center, EMS personnel transport trauma patients to the nearest medical facility that is qualified for taking care of trauma patients.

If an injury leads to death at the scene of injury, ambulance crew will contact police department and the deceased will be directly transported to the Legal Medicine Organization (LMO). LMO is the official governmental organization that deals with all deaths, including trauma fatalities. By law, police officers are required to submit a brief report to LMO officials that includes patient and injury-related characteristics. If a fatal injury occurs en route, EMS personnel transport the deceased to the hospital that was supposed to take care of the patient.

Regardless of the living status of a trauma patient, EMS personnel are required to hand in a copy of their report to the hospital authorities. After hospital arrival, all trauma fatalities regardless of their mode of arrival are required to be reported to LMO office. The report includes the death certificate and a brief description of the injury and medical treatments. If available, police reports will also accompany the hospital documents.

### Source of data

We used LMO death registry as the single largest death registry in the city. Using the computerized system of LMO, we were able to identify trauma fatalities. However in order to extract injury-related characteristics such as age, sex, intention of injury, mechanism and type of trauma and place of death occurrence (i.e. prehospital, ED or hospital), we reviewed all the medical records and legal documents (if available) of the deceased registered in LMO from September 1999 to September 2000. Since the exact time of death since injury was not recorded in the data registry, we considered the place of death as a surrogate for the three phases of trauma death. In other words we considered prehospital deaths as the Phase I, ED deaths as the Phase II and hospital deaths as the Phase III in the trimodal distribution of trauma fatalities (Figure 1). If a fatality was directly reported to LMO without a death certificate from a hospital, the place of death was determined as prehospital death. In those cases that deceased were referred by a medical care facility, often the place of death was recorded as ED death, death at operating room, death in Intensive Care Unit, etc.

The study was approved by Tehran University of Medical Sciences, medical ethics committee. For the purpose of this study, we defined children as those cases 15 years old or younger. The data was analyzed using statistical software SPSS 13.0 (Chicago, IL).

## Results

A total of 4233 trauma deaths were reported to LMO during one year of data gathering period. Children 15 years old or younger comprised approximately 10% (419 cases) of the cases. Unintentional injuries accounted for 94% (394 cases) of the fatal childhood injuries. Twenty five cases were attributed to homicide (16 cases) or suicide (9 cases). Sixty two percent of the children were male. The highest and the lowest proportion of male victims were observed in falls (78%) and poisoning (44%) -related fatalities. The mean (± SD) age of the victims was 8 (± 5) years. Children 10–15 years old comprised 42% of the injuries, followed by children 5 to 9 years old (26%) and 1 to 4 years old (25%).

Motor vehicle crashes (MVCs) (50%), burns (18%), falls (6%) and poisonings (6%) were the most common mechanism of fatal injuries. Seventy-one percent of MVC victims were pedestrians (149 cases), followed by car occupants (20%), motorcyclists (drivers and passengers) (6%) and bicyclists (3%). Table 1 summarizes the distribution of the mechanism of injury for each age group.

**Figure 2:**
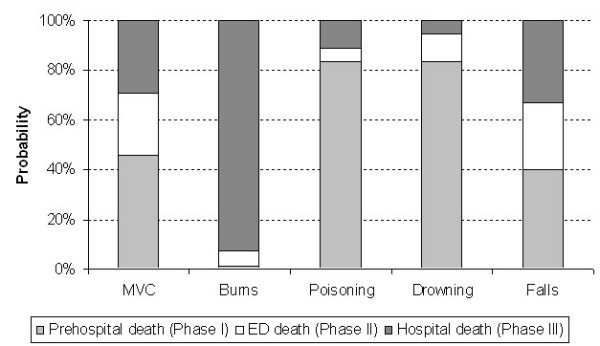
Trimodal distribution of trauma fatality for each mechanism of injury.

Evaluation of the trimodal distribution of trauma fatalities showed that in general 43% of the deaths occurred in Phase I, 20% in Phase II and 37% in Phase III. As presented in Figure 2, there was a substantial difference in this distribution among different mechanisms of injury. For example, while more than 80% of fatalities due to poisoning or drowning happened in Phase I, 92% of burn-related deaths occurred in Phase III (Figure 2).

## Discussion

Several studies have discussed the issue of fatal childhood injuries [2,10-19]. However in spite of the importance of the issue, evaluation of the trimodal distribution of fatal injuries has rarely been a topic of research in lesser developed countries [1].

Before discussing the major findings of the study, it is worth reviewing the limitations of the study. First, although LMO database is the single most complete data registry for trauma fatalities in Tehran, it does not capture all the incidents. Thus, we were not able to estimate the trauma mortality rate. Second, the time of death since injury is often not recorded in most administrative databases. Therefore, we had to use the place of death as the surrogate measurement for the time of death since injury. This issue might have led to misclassification of phase of death especially for Phase II trauma fatalities with extraordinary long prehospital times. However, Zargar et. al showed that the mean prehospital time for trauma patients in Tehran was 2 hours (SD: 2 hours) [9]. Therefore, we do not expect that such a misclassification has significantly influenced the distribution of the three phases of trauma fatality in this study [9].

Similar to some other lesser developed countries, we found that boys were more commonly affected and MVCs were the predominant mechanism of fatal childhood injuries [12-19]. Pedestrians comprised more than 70% of MVC-related fatalities which is similarly reported in other studies [20-24].

Mock et al. compared patients' outcome among three cities with different development status [1]. Table 2 summarizes the distribution of the place of death in that study. As presented, in all settings, prehospital was the predominant place of death occurrence. However, this predominance was inversely correlated with the EMS development and prehospital time in these cities. In other words, Seattle with the most advanced EMS system and the shortest prehospital time had the lowest proportion of prehospital fatalities[1]. At the same time, prehospital deaths were more common in Kumasi with no organized EMS system and the longest prehospital time. Comparison of these results with the findings form our study showed that the distribution of trauma fatalities in Tehran was more similar to such a distribution in Seattle rather than Monterrey. Differences in research methodology are the most plausible explanations for this observation.

Mock et al. excluded all fatalities that were referral from other medical care facilities [1], while these subjects were included in our analysis. Referral patients are in general sicker and at higher risk of death. Therefore, hospital trauma fatality in our study was inflated by the number of referral subjects. Unfortunately, our data does not allow us to evaluate the proportion of the patients who died in a referral hospital. However, our previous study showed that significant proportion of fatalities from MVCs or burns occurred in a few university hospitals and two burn referral hospitals that often take care of severely injured patients [9]. Second, Mock and his colleagues excluded children younger than 15 years old, which comprised the study population in our analysis. Children often play around their homes and under supervision of other adults. Therefore in the case of emergencies, they often receive a quicker response compared to adults. Furthermore, their smaller body stature makes their transportation via private vehicles easier and faster. This can explain why in our previous study children were less frequently transported to a hospital by EMS ambulances compared with adults [7]. Even among pediatric population, younger children were less probable to use EMS services relative to older children [7].

Third, Mock et al. excluded death due to burns, strangulations, hangings, or suicide by poisoning. Burns alone comprised 18% of the fatal injuries in our study, 92% of them died in a hospital.

As mentioned before, the trimodal distribution of fatal injuries has a substantial importance in resource allocation in injury control programs. For poisoning and drowning with significant proportion of trauma fatalities in Phase I, prevention is the single most important strategy for controlling the magnitude of the problem. Improvements in the quality of care often do not influence patients' outcome, since the majority of these fatalities occur before any medical care is provided to trauma victims. Injuries due to MVCs and falls often occur in Phases II and III. Under these circumstances, improvements in prehospital and ED care might significantly improve patients' outcome. For deaths that happen days or weeks after injury, improvements in hospital and ICU care might be the most important strategy in alleviating the magnitude of the problem. Burns with more than 90% hospital death in our study is the best example of this group.

We discussed the importance of prehospital and hospital care in reducing the burden of fatal injuries in Phase II and Phase III. However, we would like to emphasize that the detrimental consequences of injuries is not limited to hospital admissions and fatalities. Other physical and psychosocial consequences of injuries such as long and short term disabilities and post traumatic stress disorders might be alleviated if improvements in health care programs are associated with effective injury prevention endeavors.

Describing the different injury control strategies that could be adopted in a large city such as Tehran is beyond the scope of this paper and can be found in references [23,25-30]. However, the results of this study and previous researches in regards to the identification of the risk factors, mechanisms and severity of injuries and trauma fatality among Tehran's children [7,9] have paved the way for future interventions.

## Conclusion

Our study showed that injury prevention is the single most important solution for controlling trauma fatalities due to poisoning and drowning. Improvements in the quality of care in hospitals and intensive care units might substantially alleviate the magnitude of the problem due to burns. Improvements in prehospital and ED care might significantly decrease MVC and falls-related fatalities.

## Competing interests

The author(s) declare that they have no competing interests.

## Authors' contributions

BR and MG participated in study design and obtaining the required funding for the project; MS supervised data collection process, BR and MS conducted data analysis and prepared the first draft of the paper. BR, MS and MG have read the paper and commented on the final draft.

## Pre-publication history

The pre-publication history for this paper can be accessed here:



## Figures and Tables

**Table 1:** Distribution of the mechanism of injury for each age group

Mechanism of injury	<1	1–4	5–9	10–15	Total
MVC *					
Pedestrian	-	43 (35%)	51 (45%)	55 (31%)	149 (36%)
Car occupant	1 (11%)	5 (4%)	13 (12%)	23 (13%)	42 (10%)
Motorcyclists	-	3 (2%)	4 (4%)	5 (3%)	12 (3%)
Bicyclists	-	1 (1%)	2 (2%)	5 (3%)	8 (2%)
Burn	1 (11%)	28 (23%)	18 (16%)	27 (15%)	74 (18%)
Fall	-	7 (6%)	4 (4%)	13 (7%)	24 (6%)
Poisoning	-	11 (9%)	4 (4%)	9 (5%)	24 (6%)
Drowning	-	6 (5%)	4 (4%)	8 (5%)	18 (4%)
Others	7 (78%)	19 (15%)	10 (9%)	30 (18%)	66 (15%)

Total	9	123	110	175	417

* Motor Vehicle Crashes

**Table 2:** Comparison of the prehospital, Emergency Department (ED) and hospital deaths among USA, Mexico, Ghana and Iran

Country	Place of death occurrence		
	
	Prehospital	ED	Hospital
Seattle, USA	59%	18%	23%
Monterrey, Mexico	72%	21%	7%
Kumasi, Ghana	81%	5%	14%
Tehran, Iran	42%	20%	36%
